# Hazards of Travel—Who Will Free the Contemporary Traveler?

**DOI:** 10.3201/eid1102.AC1102

**Published:** 2005-02

**Authors:** Polyxeni Potter

**Affiliations:** * Centers for Disease Control and Prevention, Atlanta, Georgia, USA

**Keywords:** Bearden, Harlem Renaissance, collage, travel, SARS, West Nile virus, avian flu, Ebola

**Figure:**
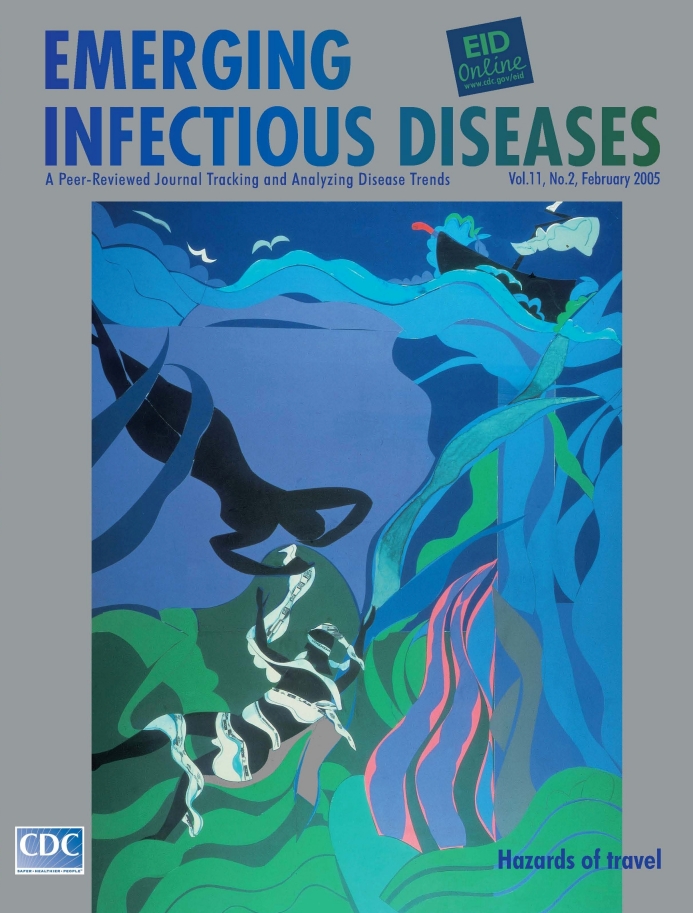
**Romare Bearden (1911–1988). The Sea Nymph (1977).** Collage on various papers with paint and graphite on fiberboard (111.8 cm x 81.3 cm). Permanent collection: Glen and Lynn Tobias. Cover art copyright Romare Bearden Foundation / Licensed by VAGA, New York, New York

A native of Mecklenburg County, North Carolina, Romare Bearden was the offspring of a middle-class family established in Charlotte, where the railroad and cotton industries flourished after the Civil War. His paternal great-grandparents, with whom he spent considerable time, were described in the 1915 publication Colored Charlotte as "former servants of Dr. Joseph Wilson the father of President Woodrow Wilson…." (*1*). His maternal grandparents, who were also influential in his development, ran a boarding house in Pittsburgh, Pennsylvania, serving migrant steel mill workers from the South.

Around 1914, Bearden's family moved north to New York and settled in Harlem. In their apartment at 154 West 131st Street, he grew up with the artistic, intellectual, and political influences of the cultural movement of the 1920s and 30s known as Harlem Renaissance. His circle included writers Langston Hughes and Ralph Ellison, musicians Duke Ellington and Fats Waller, activist W.E.B. DuBois, and artists Aaron Douglas and Jacob Lawrence (*2*). Although he spent most of his school years in New York, Bearden visited Pittsburgh often, enjoying life in his grandparents' boardinghouse, where mill workers returning from work would sit on the steps and "tell stories about down-home in the South" (*1*).

Bearden had many talents and broad academic interests. He graduated from New York University with a degree in education, but he also loved mathematics and music and was an accomplished writer and cartoonist. His editorial drawings on the social, political, and economic issues of his day (depression era soup lines, segregation, social inequality), are reminiscent of the politically charged work of Diego Rivera and other Mexican muralists and of Francisco de Goya's caprices, which chronicled the vices of 19th-century Spain. Bearden studied art throughout his life. While employed in the New York City Department of Social Services to support himself, he satisfied his growing wish to become an artist by painting during evenings and weekends. By the end of the 1930s, he was fully engaged in art.

"…[T]he function of the artist is to find ways of communicating, in sensible, sensuous terms, those experiences which do not find adequate expression in the daily round of living and for which, therefore, no ready made means of communication exists…" wrote Bearden in his first solo exhibition pamphlet in 1940 (1). In a career marked by continuous growth, he experimented with new media, always seeking the texture, form, and color that most closely embodied his artistic goals, and became one of the most creative and original artists of the 20th century.

Bearden's early work was mostly gouaches (opaque watercolors on brown paper). He became increasingly interested in the human figure but gradually moved away from representational painting toward abstraction (*3*) and "those universals that must be digested by the mind and cannot be merely seen by the eye" (*4*). By the early 1960s, he was constructing photomontages, which he continued to refine through various techniques into collages, his signature style. During the 1970s and 80s, he synthesized elements of his earlier work into an individualized art form using brown paper, brilliant color, and graphite drawings.

The collage, which dates back to medieval Persia and Japan, was known in Europe well before the 18th century and was rediscovered and used in modern times by Pablo Picasso and others. Bearden turned the medium into a narrative device, synthesizing color, form, photographic images, and patches of social commentary into intricate, richly textured, intensely emotional scenes. "…I use many disparate elements to form either a figure, or part of a background. I build my faces…from parts of African masks, animal eyes, marbles, mossy vegetation…." (*5*).

A prolific artist, Bearden painted the places where he lived and worked: the rural South, northern cities of his childhood, and the Caribbean islands where he spent the latter part of his life. His artistic goal was "to reveal through pictorial complexities the riches of a life I know." "I do not need to go looking for 'happenings,' the absurd, or the surreal," he said, "because I have seen things that neither Dalí, Beckett, Ionesco nor any of the others could have thought possible; and to see these things I did not need to do more than look out of my studio window" (*6*).

In 1976, after many years, Bearden traveled to the sites of his early childhood, only to find that everything had changed. Shortly afterwards, perhaps reflecting on his own life's journey, he embarked on a series of 20 collages based on Homer's Odyssey. Inspired by Odysseus' epic travails as he wandered the Mediterranean in search of Ithaca, these compositions showcase the essential geometry in Bearden's work. Highly finished flat panels of vivid color contain minimal surface manipulation or paint. Fluid charcoal silhouettes beneath the waves recall the dark figures adorning classical Greek pottery.

"…[T]he sparkle and pulsations of water give men and women a certain energy…" wrote Bearden in praise of his Caribbean experience (*1*), which also might have prompted this excursion into mythology. For most of us, fascination with the sea and longing for the unknown prompt travel. As the graceful nymph on this month's cover frees Odysseus from one more hurdle of his 10-year journey, we sympathize with the weary traveler. Yet, however gruesome, his impediments were imaginary—angry gods, cyclopes, sirens, Scylla and Charybdis. As we reach contemporary ports of call, the threats we meet—SARS, avian flu, West Nile virus, Ebola—are real.
